# The addition of discrimination inhibitors stimulations discrimination potential and N_2_O emissions were linked to predation among microorganisms in long term nitrogen application and straw returning systems

**DOI:** 10.3389/fmicb.2023.1337507

**Published:** 2024-01-09

**Authors:** Chunhua Jia, Guixiang Zhou, Ling Ma, Xiuwen Qiu, Jiabao Zhang, Jingkuan Wang, Congzhi Zhang, Lin Chen, Donghao Ma, Zhanhui Zhao, Zaiqi Xue

**Affiliations:** ^1^Northeast Key Laboratory of Conservation and Improvement of Cultivated Land (Shenyang), Ministry of Agriculture and Rural Affairs, College of Land and Environment, Shenyang Agricultural University, Shenyang, China; ^2^Fengqiu Experimental Station of National Ecosystem Research Network of China, State Key Laboratory of Soil and Sustainable Agriculture, Institute of Soil Science, Chinese Academy of Sciences, Nanjing, China; ^3^College of Landscape Architecture, Jiangsu Vocational College of Agriculture and Forestry, Jurong, China; ^4^School of Geomatics and Urban Spatial Informatics, Henan University of Urban Construction, Pingdingshan, Henan, China

**Keywords:** protists, ammonia-oxidizing bacteria (archaea), keystone taxa, predatory relationship, N_2_O emission, potential nitrification rate

## Abstract

**Introduction:**

Ammonia oxidizing archaea (AOA) and ammonia oxidizing bacteria (AOB) have been proven to be key microorganisms driving the ammonia oxidation process. However, under different fertilization practices, there is a lack of research on the impact of interaction between predators and AOA or AOB on nitrogen cycling at the multi-trophic level.

**Methods:**

In this study, a network-oriented microscopic culture experiment was established based on four different long-term fertilization practices soils. We used the nitrification inhibitors 2-phenyl-4,4,5,5-tetramethylimidazoline-1-oxide-3-oxyl (PTIO) and 3, 4-Dimethylpyrazole phosphate (DMPP) inhibited AOA and AOB, respectively, to explore the impact of interaction between protists and AOA or AOB on nitrogen transformation.

**Results:**

The results showed that long-term nitrogen application promoted the potential nitrification rate (PNR) and nitrous oxide (N_2_O) emission, and significantly increased the gene abundance of AOB, but had no obvious effect on AOA gene abundance. DMPP significantly reduced N_2_O emission and PNR, while PTIO had no obvious effect on them. Accordingly, in the multi-trophic microbial network, Cercozoa and Proteobacteria were identified as keystone taxa of protists and AOB, respectively, and were significantly positively correlated with N_2_O, PNR and nitrate nitrogen. However, Nitrososphaerota archaeon as the keystone species of AOA, had an obvious negative linkage to these indicators. The structural equation model (SEM) showed that AOA and AOB may be competitors to each other. Protists may promote AOB diversity through direct trophic interaction with AOA.

**Conclusion:**

The interaction pattern between protists and ammonia-oxidizing microorganisms significantly affects potential nitrification rate and N_2_O emission, which has important implications for soil nitrogen cycle.

## 1 Introduction

Soil nitrogen cycle is one of the cores of elemental cycling in soil ecosystems, which mainly includes four processes, biological nitrogen fixation, ammonification, nitrification and denitrification (Wang et al., 2021). Nitrification is an intermediate link between nitrogen fixation and denitrification (Lehnert et al., 2018). Nitrification determines the effective utilization of nitrogen by plants, but also is directly related to a series of ecological and environmental problems such as soil acidification, and greenhouse gas nitrous oxide (N_2_O) release (Aryal et al., 2022). The ammonia oxidation process is the first and rate-limiting step of nitrification, and is the central link of the global nitrogen cycle. It is mainly driven by ammonia oxidizing archaea (AOA) and ammonia oxidizing bacteria (AOB) (Zhao et al., 2020; Kaur-Bhambra et al., 2021). Previous studies of ammonia-oxidizing microorganisms affecting nitrogen transformation mainly focused on effects of abiotic factors at the same trophic level. For example, AOB was significantly correlated with potential nitrification rate (PNR) and dominated nitrification rather than AOA in calcareous soils (Zou et al., 2022). Urea nitrogen in paddy soil promoted N_2_O production by AOB more than ammonium nitrogen, and the N_2_O production by AOA was more sensitive to ammonium nitrogen (Fu et al., 2020). However, the effects of microbial interactions on nitrogen cycle under different fertilization practices and at the multi-trophic level of predation relationships (e.g., protists) have rarely been reported. Therefore, it is necessary to add discriminative inhibitors to silence the activity of AOA and AOB, in order to better explain the trophic interactions between predators and ammonia-oxidizing microorganisms.

2-phenyl-4,4,5,5-tetramethylimidazoline-1-oxide-3-oxyl (PTIO) is a stable and water-soluble organic free radical that can combine with NO to form NO_2_, and it is usually used as a nitric oxide scavenger (Lin et al., 2023). It has been reported that low concentrations of PTIO have a strong inhibitory effect on AOA rather than AOB (Sun et al., 2022). For example, under the condition of 100 μM PTIO, PTIO can inhibit the ammonia oxidation process by inhibiting AOA (Duan et al., 2018; Choi et al., 2023), and thus it was used to selectively inhibit the activity of AOA in the presence of AOB (Kits et al., 2019). 3,4-dimethylpyrazole phosphate (DMPP) is a highly soluble compound with high stability and persistent suppression effects (Li et al., 2023). Studies have shown that DMPP effectively inhibited the metabolic activity of AOB rather than AOA, which in turn led to a reduction of nitrification rate (Lei et al., 2022). Furthermore, in calcareous soils where the nitrification process was regulated by AOB, the addition of DMPP significantly suppressed N_2_O emissions (Bai et al., 2020). In general, these findings demonstrated that PTIO and DMPP can serve as effective inhibitors of AOA and AOB.

To investigate the effects of biological factors on nitrification process at the multi-trophic level, we focused on trophic interactions between protists and ammonia-oxidizing microorganisms. Protists, as predators of soil micro-food webs, can control the abundance and function of microbial species by preying on bacteria, fungi, or nematodes (Geisen et al., 2015; Geisen, 2016). Thus, the nutrient turnover rate of soil food web is increased, which ultimately regulates the decomposition of organic matter and affects nutrient cycling. Predatory behavior of protists leads to interactions between organisms within the soil microbiome to influence the response of the protist community to nitrogen application practices (Gao et al., 2019). Protists resorbed growth-limiting nutrients by feeding on bacterial cells in oligotrophic environments and ultimately stimulated the proliferation of primary producers and other bacteria (Chen et al., 2021). In addition, the interaction between protists and other microorganisms can promote the efficient utilization of organic nitrogen by AM fungal hyphae. Due to the fact that AM fungal hyphae acquire nitrogen more efficiently when both protists and prokaryotes are present in the organic nitrogen zone (Rozmoš et al., 2022). Thus, indirect (top-down control) and direct (heterotrophic) effects of protists on soil nutrient cycling perhaps increase the availability of nitrogen in the soil (Koller et al., 2013) and there is increasing attention to the role of protists in nutrient cycling. Although both protists and ammonia-oxidizing microorganisms have important implications for the soil nitrogen cycle, the effects of trophic interactions between protists and AOA or AOB on nitrification process remain unknown.

In general, understanding the effects of abiotic and biotic factors on nitrification is critically important for the regulation of soil nitrogen cycling processes. This study conducted microcosm experiments using four different long-term fertilization soils, using high-throughput sequencing technology and incorporating structural equation modeling (SEM). The aim was to (1) assess the efficacy of fertilization practices and ammonia-oxidizing microorganisms in influencing N_2_O emissions and PNR. (2) Reveal the trophic interactions between protists and AOA or AOB and their effects on nitrification process at the multi-trophic level.

## 2 Materials and methods

### 2.1 Field site and sampling

In June 2022, the soil was sampled in a long-term field experiment at Fengqiu Agricultural Ecological Experimental Station of Chinese Academy of Sciences (35°01′N, 114°32′E). The experiment has been conducted since 2010. The basic soil properties at the beginning of the experiment were shown in Table 1. This study collected soil samples from four fertilization treatments: NN (No straw and no nitrogen fertilization), CF (No straw and conventional fertilization), SM (Straw mulching and no nitrogen fertilization), SMCF (Straw mulching and conventional fertilization). NN and SM treatments were applied without nitrogen and only phosphorus and potassium fertilizers were applied. Conventional fertilization of CF and SMCF treatments indicated that both nitrogen, phosphorus and potassium fertilizers were applied. In all fertilizer treatments, the total amount of nitrogen (pure nitrogen) applied to wheat throughout the whole growth period was 210 kg ha^–1^, and the phosphorus and potassium fertilizers were 157 kg ha^–1^ for P_2_O_5_ and 105 kg ha^–1^ for K_2_O, respectively. Straw mulching was done by cutting corn straw into 20–50 mm strips after the previous season’s corn harvest, and then evenly covering the soil surface with straw after tilling (at a depth of about 15 cm). All the crop straw from each plot was returned to the same plot. The soil samples (0–20 cm depth) were collected using the five-point diagonal sampling method. Subsequently, the samples were put in sterile bags and temporarily stored in foam boxes with dry ice and taken back to the laboratory at the station within 2 h. Adequate dry ice was used to ensure that the soil samples were in an environment below 0°C. All the soil samples were passed through a 2 mm diameter mesh sieve to remove plant debris and stones, and the remaining fine root and straw residues were manually removed. The soil samples were stored at 4°C before constructing the micro world.

**TABLE 1:** Basic soil properties of the field.

	pH	Total nitrogen (g kg^–1^)	Soil organic matter (g kg^–1^)	Alkaline nitrogen (mg kg^–1^)	Total phosphorus (g kg^–1^)	Available phosphorus (mg kg^–1^)	Total potassium (g kg^–1^)	Available potassium (mg kg^–1^)
Soil properties	8.57	0.54	8.00	40.92	0.86	16.71	19.17	63.67

### 2.2 Soil microcosm experiment

Soil samples were pre-incubated at 28°C for 7 days with moisture adjusted to 50% of water holding capacity. A 21-day microcosm experiment was established by placing 20 g (equivalent dry weight) of pre-incubated soil in 120 ml serum bottles. The following treatments were set up for each fertilized soil: (i) water only (CK), (ii) urea only (U), (iii) urea + PTIO (UP), and (iv) urea + DMPP (UD). Urea was added at 200 mg N kg^–1^ dry soil and DMPP was added at 1.5% of urea N (Fan et al., 2019; Yin et al., 2021) and the concentration of PTIO was set to 100 μM, (Jung et al., 2014). Three replications were set up for all treatments and a total of 144 microcosms were established. Gas samples (20 ml) were collected from the top with a syringe at 1, 2, 3, 4, 7, 10, 14, and 21 days after incubation. N_2_O concentrations were analyzed by gas chromatograph (Agilent 7890, Agilent Technologies, Santa Clara, CA, USA). To maintain aerobic conditions, all remaining bottles were vented for 30 min after each sampling and then resealed. Soil ammonium nitrogen (NH_4_^+^-N) and nitrate nitrogen (NO_3_^–^-N) contents were determined by destructive collection of three bottles from each treatment on days 0, 7, 14, and 21 of incubation. A portion of the last sampled soil was stored at −80°C for DNA extraction.

### 2.3 DNA extraction, real-time fluorescence quantitative PCR analysis and high-throughput sequencing

Total DNA was extracted from 0.5 g of fresh Soil using the Fast DNA^®^ Spin Kit for Soil (MP Biomedicals, Inc.). The extracted DNA concentration and purity were examined by NanoDrop2000 (Thermo Fisher Scientific, Waltham, MA, USA). We used primers TAReuk454FWD1 (CCAGCASCYGCGGTA ATTCC)/TAReukREV3 (ACTTTCGTTCTTGATYRA) (Stoeck et al., 2010), Arch-amoA26F (GACTACATATTCTACACWGACT GGGC)/Arch-amoA417R (GGTGTCATATATTGGAGGCAACGT TGG) and amoA1F (GGGGTTTCTACTGGTGGT)/amoA2R(CCC CTCKGSAAAGCCTTCTTC) (Xia et al., 2011) amplified protists, AOA and AOB, respectively. AOA and AOB were analyzed on the qTOWER3/G amplification apparatus (Analytik Jena AG, Germany) with three replicates per sample. The standard curve is constructed from 8 series of diluents containing known copy number plasmids of the target gene. The reaction system for qPCR was 20 μl, including 1 μl of DNA template, 10 μl of TB Green^®^ Premix Ex Taq TM, 0.25 μl forward and 0.25 μl reverse primers, and 8.5 μl of sterilized double-distilled water. Negative control was performed using sterilized distilled water instead of the template DNA sample as the reaction template.

Amplicon sequence variants (ASVs) were generated from raw sequence data using DADA2 (Callahan et al., 2016). Briefly, after removing the primer and adapt sequences using “cutadapt” (Martin, 2011), the sequences were filtered and trimmed using “filterAndTrim” with the following parameters: maxN = 0, maxEE = c(2,2), truncQ = 2. Chimeric sequences were detected and removed using “removeBimeraDenovo” based on the “consensus” method. The taxonomy assignment for protist was performed with the “assignTaxonomy” function (minimal bootstrap value 50) based on the PR2 database (V4.14.0) (Guillou et al., 2012). Taxonomy of the amoA ASVs was assigned using BLASTn based on the nucleotide database in NCBI with the default algorithm parameters (expect threshold was 0.05, word size was 28, match/mismatch scores were 1, -2, gap costs was linear, and e-value was 0.00001) (Kõljalg et al., 2005). The unassigned ASVs were filter for further study. The ASVs of 18S rRNA assigned as Rhodophyta, Streptophyta, Metazoa, Fungi, and unidentified Opisthokonta were removed for protist community analysis. All high-throughput sequencing steps were performed by Guangdong Magi Gene Technology Co., Ltd. (Guangzhou, China).

### 2.4 Statistical analysis

One-way analysis of variance (ANOVA) in SPSS Statistics 21 was used to evaluate the effects of different treatments on N_2_O emission and copy number of ammonia-oxidizing microorganisms. The “vegan” package in R v4.2.1 software was used to conduct principal coordinate analysis (PCoA) (Anderson and Willis, 2003). Pearson correlation analysis was conducted using R v4.2.1 software packages reshape2 and psych to examine the correlation between key taxa and soil physicochemical properties, as well as between key taxa. A heat map was generated using the “pheatmap” package.

The co-occurrence network analysis based on Spearman’s correlation matrix was calculated in the “Hmisc” package in R v4.2.1 (Wang et al., 2022). ASVs with a sum of relative abundance higher than 90% were selected, and then two correlations between all ASVs were calculated by combining protists (744 PASVs), AOA (105 ASVs) and AOB (67 BASVs) into one relative abundance table. To distinguish different microorganisms, we used PASV to represent the ASV of protists and BASV to represent the ASV of AOB. A correlation threshold higher than 0.6 and a *P*-value lower than 0.01 are considered to be stable co-occurrence relationships. Adjustments were made by using test corrections from the Benjamini-Hochberg procedure to reduce the chances of obtaining false positive results (Benjamini and Hochberg, 1995). The co-occurrence network was visualized and analyzed by “Gephi” software (Bastian et al., 2009). Subsequently, the connections within modules (*Zi*) and between modules (*Pi*) were calculated using the package “igraph” in R v4.2.1. According to the topological characteristics of nodes, node attributes were classified into four types, including: Module hubs, nodes with high connectivity within a module (*Zi* > 2.5 and *Pi* < 0.62). Connectors, nodes with high connectivity between two modules (*Zi* < 2.5 and *Pi* > 0.62). Network hubs, nodes with high connectivity throughout the network (*Zi* > 2.5 and *Pi* > 0.62), and Peripherals, nodes that do not have high connectivity both within and between modules (*Zi* < 2.5 and *Pi* < 0.62) (Deng et al., 2016). Moreover, according to Banerjee et al. (2018) and Shi et al. (2020), module hubs connectors, and network hubs were defined as keystone species in this study. Among them, the taxa of module hubs generally mediate interactions between species within the module and have significant implications for module function by regulating energy and material exchange within the module (Guimera and Nunes Amaral, 2005; Toju et al., 2018). Therefore, this study mainly focused on module hubs.

The SEM quantified the direct and indirect effects of soil physicochemical properties, protists, AOA, and AOB on PNR and N_2_O. Soil physicochemical properties (soil NO_3_^–^-N and NH_4_^+^-N) and microbial diversity (protists, AOA and AOB diversity) were represented by PCoA data of corresponding indicators. The maximum likelihood estimation method was applied to fit the model, and the parameters were consistent with relatively low chi-square and *P* > 0.05, goodness fit index (GFI) > 0.90 and root mean square error of approximation (RMSEA) < 0.05 (Grace and Keeley, 2006). All SEM analyses were performed using AMOS 21.

## 3 Results

### 3.1 Changes in N_2_O emission rates and PNR

**FIGURE 1:**
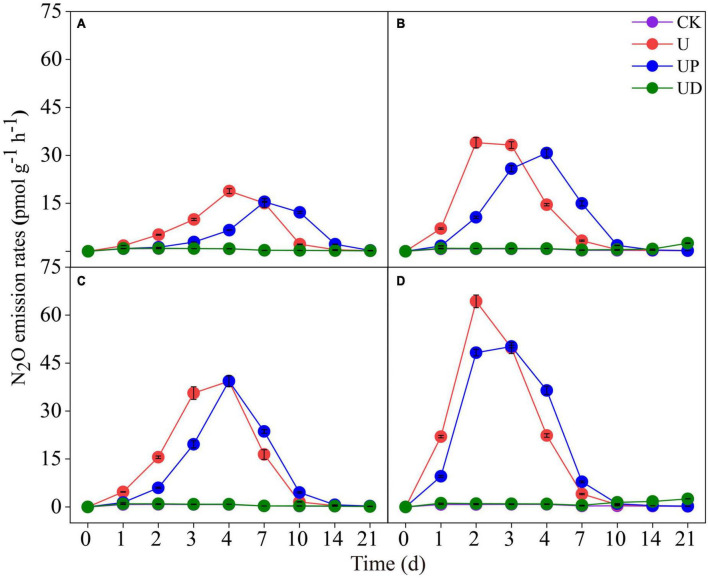
Dynamic of N_2_O emission rates in the four fertilization practices from NN **(A)**, CF **(B)**, SM **(C)** and SMCF **(D)** under four treatments, including CK, U, UP and UD during the experimental incubation. Vertical bars indicate standard errors of the means (*n* = 3). NN, no straw and no nitrogen fertilization; CF, no straw and conventional fertilization; SM, straw mulching and no nitrogen fertilization; SMCF, straw mulching and conventional fertilization. CK, water only; U, urea only; UP, urea + PTIO; UD, urea + DMPP.

Nitrous oxide emission rate was significantly affected by soil fertilization practices and nitrification inhibitors. For the urea (alone) treatments, the N_2_O emission rate of the long-term no-nitrogen treatments (NN, SM) peaked on day 4 (Figures 1A, C), and the long-term nitrogen treatments (CF, SMCF) reached the maximum rates on day 2 (Figures 1B, D). Meanwhile, long-term nitrogen and straw treatment (SMCF) had the highest N_2_O emission rate (Figure 1D). N_2_O emissions ranged from 18.84∼64.32 pmol g^–1^ h^–1^ under different fertilization practices, thus, long-term nitrogen and straw application promoted N_2_O emission by 3.4 times. The N_2_O emission rate was reduced at different degrees by the application of nitrification inhibitors. In all soils, N_2_O emission rates of U and UP treatments rapidly increased during the first week of incubation. During the whole incubation period, the UD treatment significantly decreased the N_2_O emission rate, and the emission trend was essentially consistent with CK. The range of N_2_O emissions under nitrification inhibitor treatments was 1.03∼64.32 pmol g^–1^h^–1^, which reduced N_2_O emissions by 62.4 times. In contrast, nitrification inhibitors were much more effective on N_2_O emissions than fertilizer treatments. Additionally, compared with U treatment, NO_3_^–^-N increased slowly in the UD treatment, and the UP treatment reduced NH_4_^+^-N content (Supplementary Figure 1).

The effect of soil fertilization practices on PNR was significant only in the CK treatment, i.e., the long-term nitrogen application treatments (CF and SMCF) obviously increased PNR under the condition of no exogenous urea addition. Moreover, long-term nitrogen and straw application promoted PNR by 7.5 times. However, the addition of urea during incubation reduced the differences of soil fertilization practices. The U and UP treatments had the highest PNR in all soils. Compared with U treatment, the PNR was significantly increased in UP treatment on day 7 of NN and CF treatments, while there was a decreasing trend on the day 14 and 21. The PNR of UP treatment decreased with the extension of incubation time compared to the U treatment in SM treatment. The UD treatment significantly decreased PNR, with a trend consistent with CK or even obviously lower than CK (Table 2). PNR was reduced by 80 times after treatment with nitrification inhibitors. In general, the effect of fertilization practices on PNR was much lower than that of nitrification inhibitors.

**TABLE 2:** PNR during incubation of four nitrification inhibitors treatments (CK, U, UP, UD) within four fertilization practices (NN, CF, SM, SMCF).

Treatments	Time	CK	U	UP	UD
NN	7 d	0.056 ± 0.003c	0.605 ± 0.017b	0.821 ± 0.037a	0.004 ± 0.0002c
	14 d	0.071 ± 0.003c	0.185 ± 0.003b	0.234 ± 0.006a	0.003 ± 0.0002d
	21 d	0.061 ± 0.003b	0.118 ± 0.004a	0.116 ± 0.006a	0.004 ± 0.0002c
CF	7 d	0.117 ± 0.001c	0.232 ± 0.012b	0.647 ± 0.014a	0.007 ± 0.0003d
	14 d	0.180 ± 0.008b	0.222 ± 0.011a	0.230 ± 0.009a	0.010 ± 0.0002c
	21 d	0.186 ± 0.008c	0.238 ± 0.014b	0.325 ± 0.014a	0.024 ± 0.001d
SM	7 d	0.065 ± 0.003c	0.525 ± 0.021b	1.331 ± 0.033a	0.006 ± 0.0003c
	14 d	0.092 ± 0.002b	0.191 ± 0.007a	0.192 ± 0.005a	0.005 ± 0.0003c
	21 d	0.079 ± 0.003c	0.159 ± 0.005a	0.142 ± 0.007b	0.004 ± 0.0002d
SMCF	7 d	0.156 ± 0.005b	0.308 ± 0.008a	0.287 ± 0.014a	0.007 ± 0.0004c
	14 d	0.234 ± 0.011b	0.288 ± 0.007a	0.230 ± 0.011b	0.009 ± 0.0002c
	21 d	0.278 ± 0.009b	0.323 ± 0.010a	0.331 ± 0.008a	0.030 ± 0.001c

Different letters in the same row denote significant differences at *P* < 0.05 level.

### 3.2 Changes in gene abundance and analysis of microbial community structure

**FIGURE 2:**
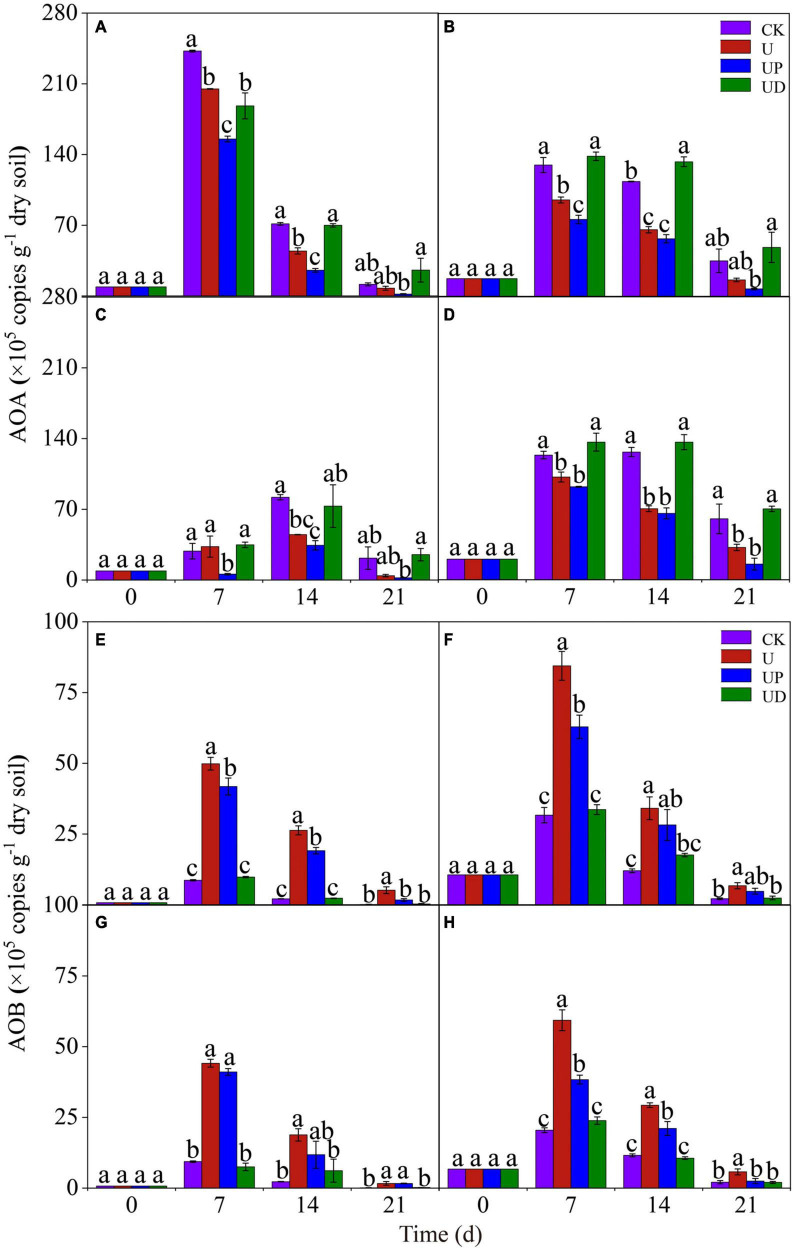
Dynamic of the AOA **(A–D)** and AOB **(E–H)** gene copies in the four fertilization practices from NN **(A,E)**, CF **(B,F)**, SM **(C,G)**, and SMCF **(D,H)** under four treatments, including CK, U, UP, and UD during the experimental incubation. Vertical bars indicate standard errors of the means, different lowercase letters represent significant differences within groups (*n* = 3). NN, no straw and no nitrogen fertilization; CF, no straw and conventional fertilization; SM, straw mulching and no nitrogen fertilization; SMCF, straw mulching and conventional fertilization; CK, water only; U, urea only; UP, urea + PTIO; UD, urea + DMPP.

To explore the effects of fertilization practices and nitrification inhibitors on the activity of ammonia-oxidizing microorganisms, we analyzed the changes of AOA and AOB gene abundance. For AOA, the highest gene abundance of all soil treatments was found in the NN treatment on day 7. Moreover, the UP treatment reduced AOA gene abundance in all soils. Unexpectedly, obviously higher AOA gene abundance was observed in UD and CK treatments than in U treatment (Figures 2A–D). For AOB, CF treatment had the highest gene abundance on day 7. It is noteworthy that the gene abundance of SMCF treatment was lower than that of CF treatment. The effect of nitrification inhibitors on the AOB gene abundance under different fertilization practices showed the same trend. AOB gene abundance was significantly improved in U treatment and obviously decreased in UD treatment for all soils (Figures 2E–H). It appeared that AOA was better suited to oligotrophic environments, while AOB was more active in nitrogen-rich environments.

**FIGURE 3:**
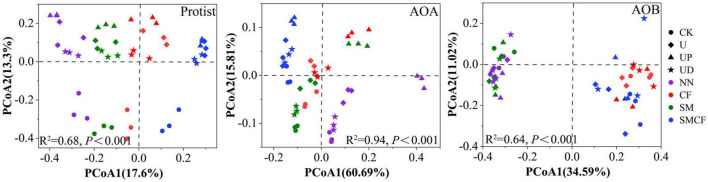
The PCoA analysis of protists, AOA and AOB within four fertilization practices (NN, CF, SM, SMCF) under four treatments, including CK, U, UP, and UD. NN, no straw and no nitrogen fertilization; CF, no straw and conventional fertilization; SM, straw mulching and no nitrogen fertilization; SMCF, straw mulching and conventional fertilization; CK, water only; U, urea only; UP, urea + PTIO; UD, urea + DMPP.

We further analyzed the response of microbial community structure to fertilization practices and nitrification inhibitors. Soil fertilization practices and nitrification inhibitor treatments had different effects on the community structure of protist, AOA, and AOB. Notably, all microbial communities in NN and SMCF treatments showed obvious separation, suggesting that long-term nitrogen and straw application significantly changed the microbial community structure (Figure 3). For protists, CK treatment was significantly detached from other treatments, while there was no obvious variation in community structure between U, UP, and UD treatments. For AOA, significant changes in community structure occurred in the UP treatment. Compared with nitrification inhibitor treatments, different fertilization soils had a greater impact on AOB community structure. For example, the AOB community structure in soils with long-term non-nitrogen application was clearly apart from soils with long-term nitrogen application.

### 3.3 Microbial interaction networks and key taxa analysis under nitrification inhibitor treatments

**FIGURE 4:**
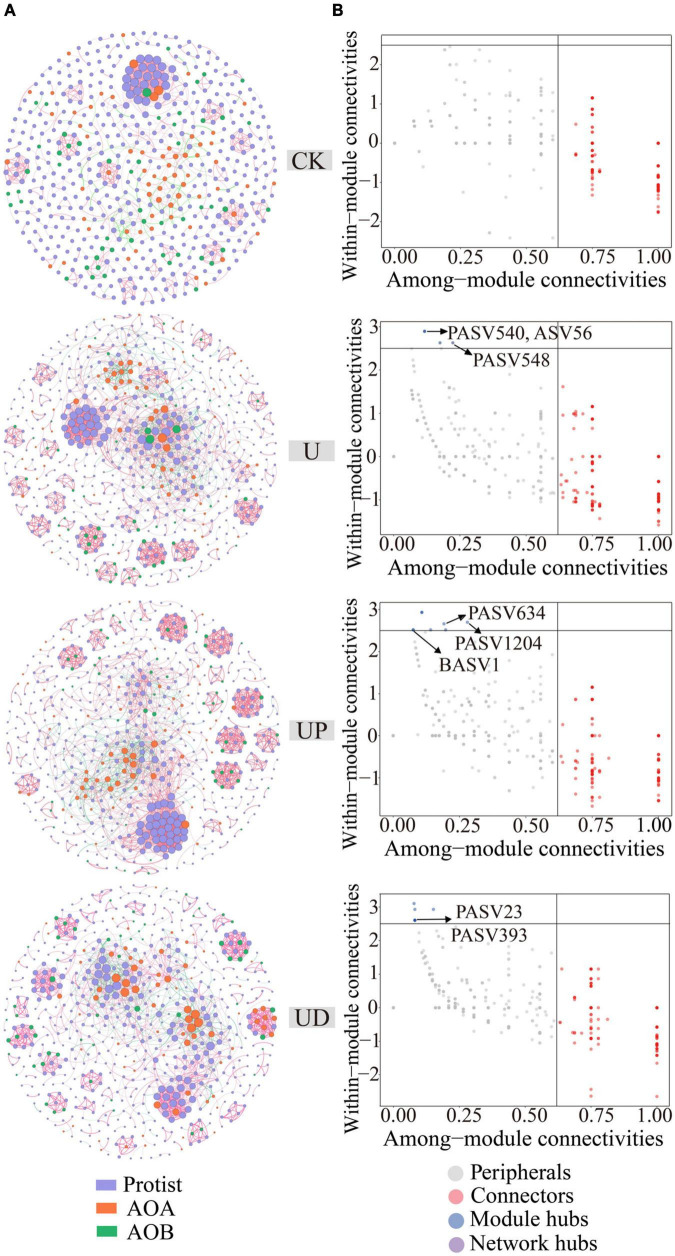
The co-occurring network of protist, AOA and AOB under four treatments, including CK, U, UP and UD **(A)**. Classification of nodes to identify keystone species within the networks **(B)**. NN, no straw and no nitrogen fertilization; CF, no straw and conventional fertilization; SM, straw mulching and no nitrogen fertilization; SMCF, straw mulching and conventional fertilization; CK, water only; U, urea only; UP, urea + PTIO; UD, urea + DMPP.

**FIGURE 5:**
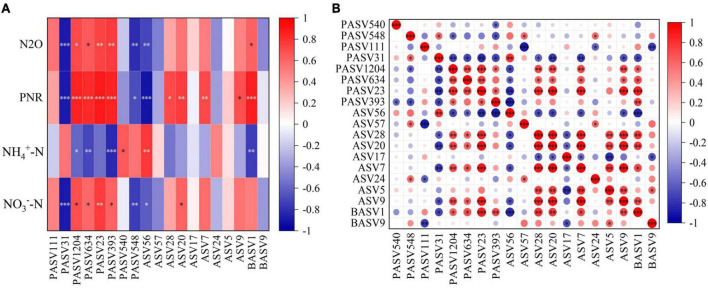
Pearson correlations between key taxa and physicochemical properties **(A)** and within key taxa **(B)**. *P < 0.05, **P < 0.01, ***P < 0.001. NN, no straw and no nitrogen fertilization; CF, no straw and conventional fertilization; SM, straw mulching and no nitrogen fertilization; SMCF, straw mulching and conventional fertilization; CK, water only; U, urea only; UP, urea + PTIO; UD, urea + DMPP.

In order to determine the impact of nitrification inhibitors on the symbiotic relationship of soil microorganisms, the co-occurring networks of protists, AOA, and AOB were constructed for CK, U, UP, and UD treatments, respectively, and the topological parameters of each network were calculated (Figure 4A). Compared to CK, the network nodes, edges, average degree, clustering coefficient and network density were increased for all urea-added treatments. Notably, all topological properties of UD treatment were reduced, resulting in a lower complexity of the network (Supplementary Table 1). Meanwhile, the direct microbial interaction showed the lowest positive correlation ratio (79.92%) under UD treatment. Nodes with higher within module connectivity (Module hubs, *Zi* > 2.5 and *Pi* < 0.62) were identified as key taxa in the network. These taxa may play an important role in maintaining the stability of network structure. There were no module hubs in CK treatment (Figure 4B), while UP and UD treatments had more key taxa than U treatment. The key taxa identified in the U, UP, UD treatments were mainly Cercozoa, Lobosa, Sagenista, Crenarchaeota and Proteobacteria (Supplementary Table 2). Moreover, the relative abundances of key taxa were also highly correlated with physicochemical properties associated with nitrogen transformation (Figure 5).

N_2_O, PNR, NO_3_^–^-N, and NH_4_^+^-N are indicators closely related to the nitrification process. However, in contrast to the other indicators, NH_4_^+^-N was mainly negatively correlated with key species of microorganisms. Among the key taxa of the protists, the proportion of key taxa with significant positive correlations with N_2_O, PNR and NO_3_^–^-N was 50%, while the proportion of negative correlations was only 25%. The key taxa of protists with positive correlations were PASV1204, PASV634, PASV23, and PASV393, classified at the phylum level as Cercozoa, Lobosa and Sagenista. For AOA, only ASV56 was strongly linked to the four physicochemical properties, but the correlation was mainly negative. For AOB, although there were only two key taxa, BASV1 was positively associated with both N_2_O and PNR, and belonged to the Proteobacteria at the phylum level (Figure 5A). Additionally, the proportion of key species of AOA that were significantly negatively related to PASV1204, PASV634, PASV23, and PASV393 was 11%, and the proportion of positively correlated key species was 50% for AOB. For AOA, only ASV56 was significantly negatively linked to key species of protists, and BASV1 of AOB had the opposite pattern to ASV56 (Figure 5B).

### 3.4 Effects of microbial interactions on PNR and N_2_O under nitrification inhibitor treatments

**FIGURE 6:**
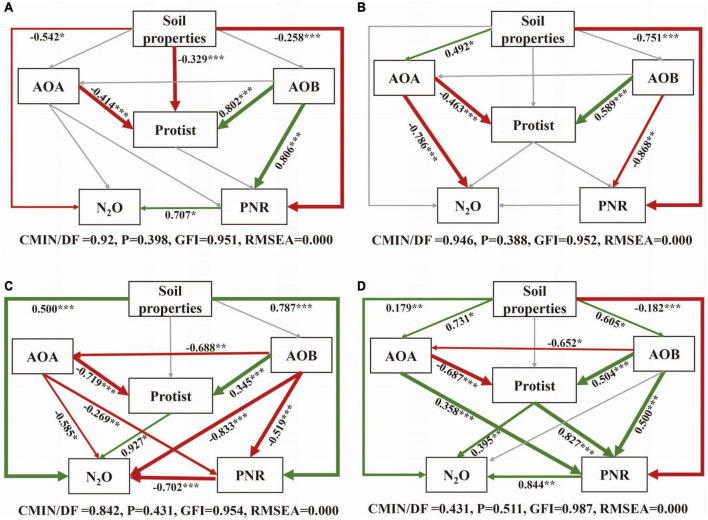
Structural equation model (SEM) analysis of the relationships between soil properties, protist, AOA, AOB and N_2_O and PNR under CK **(A)**, U **(B)**, UP **(C)**, UD **(D)** treatments. The arrow width is proportional to the strength of the relationship. Green edge: positive correlation; Red edge: negative correlation. Adjacent numbers in the same direction as the arrow indicate the correlation coefficient. Paths with non-significant coefficients are shown as gray lines. The significance levels are indicated by *P < 0.05, **P < 0.01, ***P < 0.001. NN, no straw and no nitrogen fertilization; CF, no straw and conventional fertilization; SM, straw mulching and no nitrogen fertilization; SMCF, straw mulching and conventional fertilization; CK, water only; U, urea only; UP, urea + PTIO; UD, urea + DMPP.

The SEM was constructed to further clarify the relationship between soil properties, microorganisms, PNR and N_2_O under different nitrification inhibitor treatments (Figure 6). In all treatments, AOA had a direct negative effect on protists, conversely, AOB had a direct positive impact on protists. In CK and U treatment, the influences of microorganisms on PNR and N_2_O were mostly insignificant (Figures 6A, B). Microorganisms were more closely related to each other and to PNR and N_2_O with the addition of nitrification inhibitors (Figures 6C, D). In terms of standardized total effects, the addition of nitrification inhibitors resulted in a greater contribution of AOA and AOB on protist compared to the U treatment. To be specific, the interaction between AOB and protists increased the contribution to N_2_O after inhibiting AOA, the suppression of AOB enhanced the contribution of interaction between AOA and protists to PNR (Supplementary Figure 2). Overall, when AOA was suppressed, only N_2_O was significantly affected by interaction between AOB and protist. The interaction between AOA and protists obviously affected both N_2_O and PNR with high contributions when AOB was inhibited.

## 4 Discussion

### 4.1 Effects of soil fertilization practices and nitrification inhibitors on N_2_O emission and PNR

The highest N_2_O emissions occurred in treatment with long-term nitrogen and straw application (Figure 1), which is consistent with recent studies (Xia et al., 2018). Long-term nitrogen application stimulates N_2_O emissions by affecting biological processes such as nitrification and denitrification in soil (Wu et al., 2018; Bai et al., 2020). Meanwhile, straw application can alleviate the limitation of soil N_2_O emission by providing carbon and nitrogen sources. In particular, straw addition enhances the availability of unstable carbon, which provided energy for the growth of N_2_O-producing microorganisms (e.g., AOA and AOB) in the soil (Wu et al., 2020).

As is well known, N_2_O is an important byproduct of nitrification or denitrification (Ma et al., 2012). The ammonia oxidation process driven by AOA and AOB is the initial and rate limiting step of nitrification (Hu and He, 2017; Lin et al., 2020). Our study confirmed that N_2_O emission was slightly reduced when AOA was inhibited and significantly reduced when AOB was suppressed (Figure 1). It indicated that AOB may be the main driver of N_2_O emission during the nitrification process in this study. Firstly, nitrification denitrification and incomplete NH_2_OH oxidation are the causes of N_2_O production by AOB in alkaline soils (Wu et al., 2018). Secondly, AOA seems to lack nitrification-denitrification ability due to the absence of NO reductase (Walker et al., 2010). Therefore, the effect of suppressing AOA on N_2_O emissions was not obvious. Except for N_2_O, PNR is also an important chemical index to characterize the nitrification of aerobic ammoxidation microorganisms, which can be used to quantify nitrification potential. Our study revealed that long-term nitrogen application significantly increased PNR, while PNR was reduced sharply after AOB was suppressed (Table 2). On the one hand, long-term nitrogen application provided sufficient substrate for nitrifying microorganisms and increased microbial activity (Wang et al., 2019), which in turn improved PNR. On the other hand, suppression of AOB reduced PNR probably owing to the fact that AOB is the major contributor to nitrification potential in neutral and alkaline soils (Ren et al., 2023). Thus, inhibition of AOB by DMPP indirectly suppressed PNR.

### 4.2 Effects of soil fertilization practices and nitrification inhibitors on AOA and AOB gene abundance

In this study, the long-term no nitrogen treatment had the highest AOA gene abundance (Figure 2A). This can be explained by the fact that AOA is dominant in nitrogen-limited environment (Wang et al., 2015), and long-term non-nitrogen soils provide favorable conditions for AOA to compete for ecological niches. In contrast to AOA, the peak of AOB gene abundance appeared in long-term nitrogen treatment (CF). It is due to that AOB is suited to survive in alkaline and nitrogen-enriched environments, and the application of nitrogen can provide high soil NH_4_^+^-N concentration for AOB growth (Verhamme et al., 2011; Sun et al., 2023). Additionally, we found that AOB gene abundance was lower in SMCF treatment than in CF treatment (Figure 2H). The incorporation of straw increased soil C/N, thereby delaying the nitrification process (Elrys et al., 2021; Wang et al., 2023), and thus the decrease of AOB gene abundance is understandable.

The addition of nitrification inhibitors had different effects on AOA and AOB. Our results showed that PTIO significantly inhibited the AOA gene abundance (Figures 2A–D). This may be explained by the high sensitivity of AOA to PTIO (Martens-Habbena et al., 2015). Interestingly, AOA gene abundance was obviously increased after suppressing AOB in long-term nitrogen fertilization soils (Figures 2A–D). This was inconsistent with the results of most studies that revealed AOA populations were unchanged or decreased (Di and Cameron, 2011). Firstly, there may be substrate competition between AOA and AOB, and the suppression effect of DMPP may free AOA from the competition of AOB (Fan et al., 2019). Secondly, DMPP, as an organic compound, may serve as a useful C-substrate to promote AOA growth (Florio et al., 2014). Meanwhile, the application of DMPP strongly inhibited the AOB gene abundance (Figures 2E–H) in all soils. It was worth noting that AOA activity was not affected by DMPP (Figures 2A–D). In fact, the different responses of AOA and AOB to DMPP inhibition may be attributed to different nitrifying enzyme systems. The AOA amoA sequence is more similar to the gene encoding bacterial micromethane monooxygenase (pMMO), resulting in functional differences between the AMO of AOA and AOB (Jung et al., 2014). The nitrification inhibitor DMPP decreases AMO activity, whereas MMO is unaffected (Weiske et al., 2001). Therefore, DMPP specifically inhibited AOB activity.

### 4.3 Interactions between protists and AOA or AOB

In contrast to AOA and AOB, protists are significantly affected by nitrogen fertilizer rather than inhibitors. Protists are the predators of soil micro-food web and the response to nitrogen are sensitive (Zhao et al., 2021). To explore the effect of interactions between protists and AOA or AOB on nitrogen transformation, a symbiotic network between them was constructed. We found that the addition of urea increased the complexity of the soil microbiome network and caused closer network connectivity (Supplementary Table 1). As nitrogen addition increased the availability of resources and food in the soil, it enhanced the interactions within the microbiome to improve resource utilization efficiency (Shi et al., 2016; Yu et al., 2018). Remarkably, the network complexity was lower when AOB was suppressed by DMPP. This may be due to the key taxa of AOB was inhibited, which can maintain network stability and increase network complexity. It was also confirmed by the fact that AOB was not identified in the keystone species treated with UD (Figure 4).

Nodes with high connectivity within the module (*Zi* > 2.5 and *Pi* < 0.62) were identified as key taxa. These taxa may function through energy and substance exchange within the module (Deng et al., 2016). When they are removed or disappeared from a particular ecosystem, it can lead to drastic changes in that ecosystem (Ikegami, 2005). In this study, the key species of protists after inhibitor addition mainly included PASV1204, PASV634, PASV23, and PASV393 (Supplementary Table 2), all of which were significantly positively correlated with N_2_O, PNR and NO_3_^–^-N. We further classified the key species and determined that they belong to Cercozoa, Lobosa and Sagenista at the phylum level. However, only Cercozoa was the protist species that appeared consistently since suppressing AOA or AOB, which was classified as generalist in microbial networks (Barberán et al., 2012). It emphasized the important role in microbial symbiosis. Cercozoa belongs to the predatory protist in the functional classification. It generally affects soil nutrient mineralization by top-down regulation of microbial biomass and activity and by affecting the acquisition of soil nitrogen and carbon enzyme activities (Wei et al., 2021; Wu et al., 2022). Additionally, BASV1, belonging to Proteobacteria at the phylum level, is the only AOB key species identified after inhibiting AOA. It is strongly positively associated with N_2_O, PNR, and NO_3_^–^-N, and key species of protist (Figure 5). It is well known that Proteobacteria is a key denitrification species with the potential to produce and reduce N_2_O (Xiang et al., 2023).

For AOA, on the one hand, N_2_O and PNR showed no significant changes or increased trends after AOA was inhibited. Meanwhile, we found that only ASV56 was significantly negatively correlated with both N_2_O and PNR among the key species of AOA. On the other hand, only ASV56 was negatively linked to keystone species of protists. Furthermore, the SEMs of four treatments showed a significant negative correlation between AOA and protists. The standardized total effects also indicated high negative contributions of AOA to protists. Therefore, we speculated that among the key species of AOA, ASV56 may play a major role in microbial interactions in this study. Since the key taxa of AOA was not identified, the relevant functions were analyzed by NCBI similar sequence alignment (Supplementary Table 3). By comparison, the key AOA species may be Nitrososphaerota archaeon, which can obtain energy during ammonia oxidation and utilize carbon dioxide as a chemoautotrophic carbon source (Wei et al., 2023). The interactions between protists and AOA or AOB may provide substrates and motivation for the soil nitrification process and stimulate nitrogen transformation. Specifically, Cercozoa had a higher C/N than sub-trophic level organisms, and they excreted excess nitrogen making it available to other microorganisms (Jousset, 2017). Meanwhile, Proteobacteria and Nitrososphaerota archaeon can also use the nitrogen secreted by protists to promote PNR and the N_2_O emission. Recent studies have found that the symbiotic relationship between Cercozoa and Proteobacteria was closely related to soil nitrogen transformation process (Xiao et al., 2022). The predator-prey interactions between Cercozoa and Thaumarchaeota increased nutrient turnover (Gu et al., 2023). These all support the above view.

The SEM analysis also provided a basis for the above argument. Protists were found to be the highly negatively correlated with AOA and significant positively associated with AOB. The addition of nitrification inhibitors resulted in a strong negative linkage between AOA and AOB. At the same time, standardized total effects indicated that AOA or AOB was closely related to protists. When one of AOA and AOB was inhibited, the other interacted with protists to increase contributions on N_2_O or PNR. Since the fact that both AOA and AOB use ammonia nitrogen as energy substrates, there is a direct nutritional competition between them (French et al., 2021; Sun et al., 2022). When one of the microorganisms was suppressed, the interaction between the other and protists may be more closely. Thus, the addition of nitrification inhibitors may promote the interactions between ammonia-oxidizing microorganisms and protists, and the trophic interaction may stimulate nitrogen turnover (Gao et al., 2019). Therefore, the addition of nitrification inhibitors is a noteworthy agricultural strategy to improve the soil nitrogen cycle.

## 5 Conclusion

Both abiotic and biotic factors are important factors affecting nitrogen transformation indicators N_2_O and PNR. The highest N_2_O emissions in different soil fertilization practices were found in long-term nitrogen and straw application treatment, and the PNR was also significantly increased in the treatment of long-term nitrogen application. In addition, our results demonstrated that the addition of PTIO and DMPP significantly inhibited the activity of AOA and AOB, respectively, and AOB was the main microorganism driving N_2_O emissions and stimulating PNR in alkaline fluvo-aquic soil. Finally, the relationship between keystone taxa and N_2_O and PNR in the network directly reflected the relevance of corresponding microorganisms with these indicators. Protists were significantly negatively associated with AOA and positively linked to AOB. There may be competition between AOA and AOB, and protists may promote the diversity of AOB through direct trophic interaction with AOA, thereby affecting the nitrogen transformation process. In the future, we can regulate the interaction between protists and ammonia oxidizing microorganisms by modulating key taxa of protists, AOA and AOB, and finally achieve the goal of managing soil nitrogen cycle in the field.

## Data availability statement

The datasets presented in this study can be found in online repositories. The names of the repository/repositories and accession number(s) can be found below: NCBI–PRJNA1035458, PRJNA1035811, and PRJNA1035828.

## Author contributions

CJ: Data curation, Formal analysis, Investigation, Methodology, Software, Validation, Visualization, Writing—original draft, Writing—review and editing. GZ: Conceptualization, Validation, Supervision, Writing—review and editing. LM: Writing—review and editing. XQ: Writing—review and editing. JZ: Writing—review and editing, Conceptualization, Funding acquisition, Project administration, Resources, Validation. JW: Methodology, Writing—review and editing. CZ: Methodology, Writing—review and editing. LC: Resources, Writing—review and editing. DM: Resources, Writing—review and editing. ZZ: Data curation, Writing—review and editing. ZX: Data curation, Writing—review and editing.
