# Supplementary material for: The addition of discrimination inhibitors stimulations discrimination potential and N2O emissions were linked to predation among microorganisms in long term nitrogen application and straw returning systems
Source: Front Microbiol. 2024 Jan 9;14:1337507. doi: 10.3389/fmicb.2023.1337507 (PMC10803610; doi:10.3389/fmicb.2023.1337507)
Supplement: Supplementary file 1 [file Data_Sheet_1.docx]

Supplementary Material

Supplementary Material

1. Figure S1
2. Figure S2
3. Table S1
4. Table S2
5. Table S3
6. References

**Supplementary Figures**


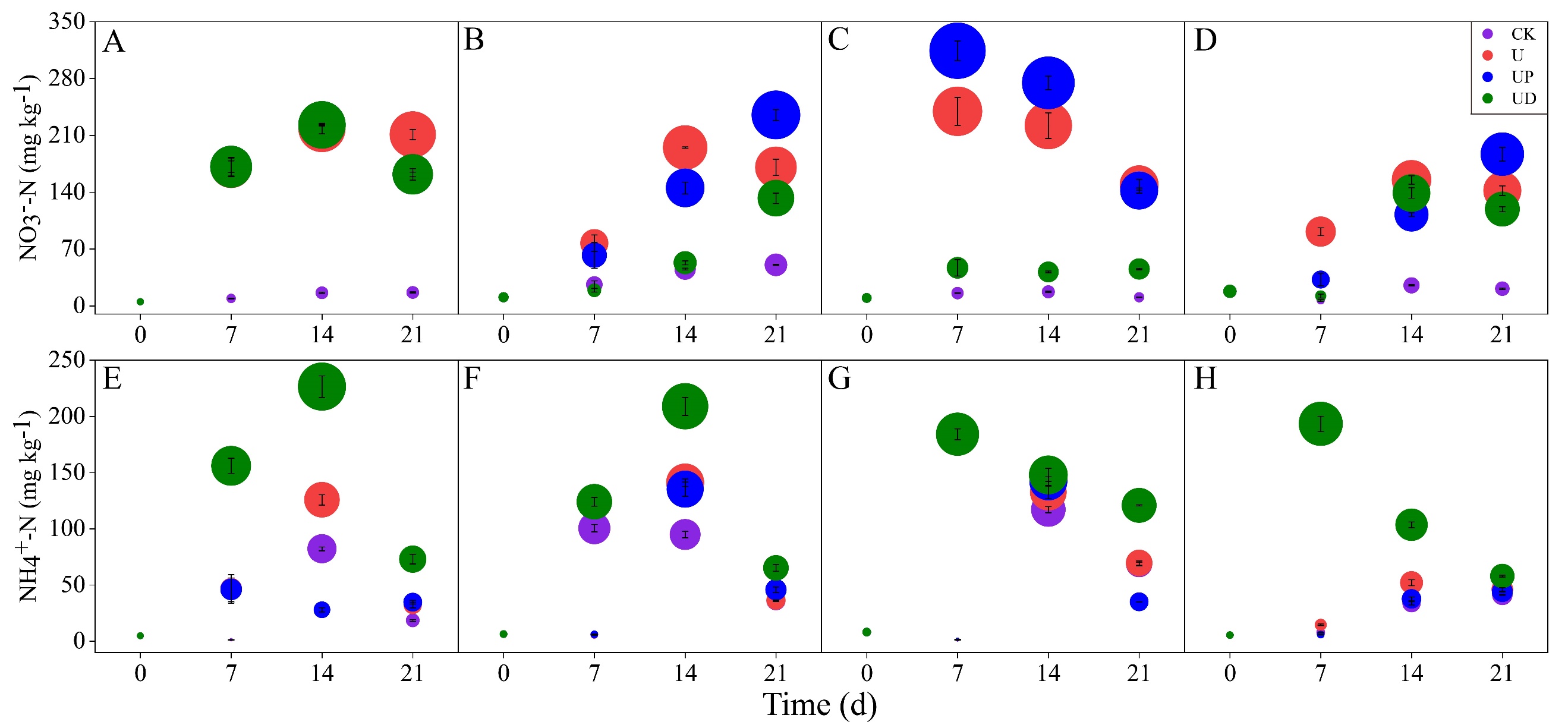


**Supplementary Figure 1.** The dynamics of NO_3_^-^-N (A–D) and NH_4_^+^-N (E–H) concentrations in the soils from NN (A, E), CF (B, F), SM (C, G) and SMCF (D, H) under four treatments, including CK, U, UP and UD during the experimental incubation. Vertical bars indicate standard errors of the means (n = 3).


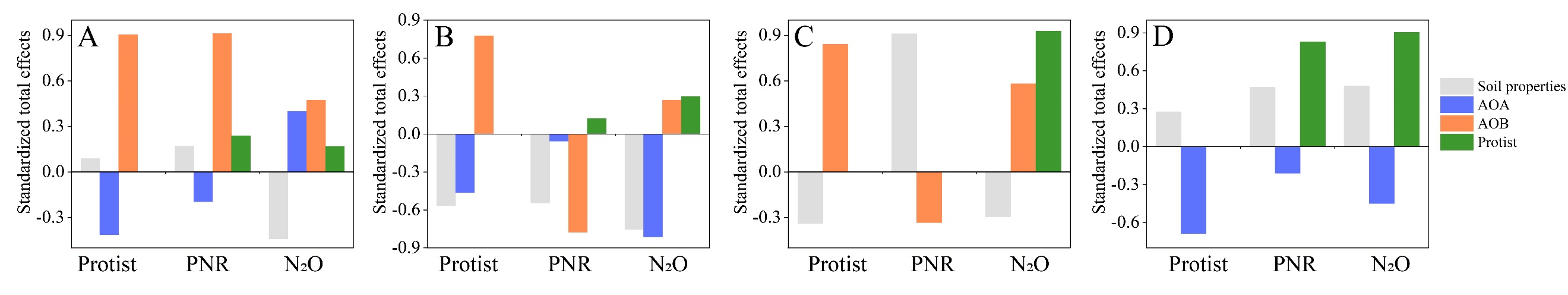


**Supplementary Figure 2.** Standardized total effects of AOA and AOB on protists and soil properties, as well as the three microorganisms on PNR and N_2_O under CK (A), U (B), UP (C) and UD (D) treatments

**Supplementary Tables**

**Supplementary Table 1.** Topological properties of co-occurring networks for four treatments (CK, U, UP, UD)

| Treatment | CK | U | UP | UD |
| --- | --- | --- | --- | --- |
| Number of nodes | 539 | 639 | 641 | 629 |
| Number of edges | 1106 | 2041 | 2326 | 1753 |
| Average degree | 4.104 | 6.388 | 7.257 | 5.574 |
| Percentage of positive (negative) correlations (%) | 92.22 (7.78) | 86.28 (13.72) | 87.83 (12.17) | 79.92 (20.08) |
| Clustering coefficient | 0.626 | 0.658 | 0.635 | 0.619 |
| Network density | 0.008 | 0.01 | 0.011 | 0.009 |

**Supplementary Table 2.** The classification of key taxa identified by the microbial network

| Treatments | Key taxa | Kingdom | | Phylum | Class | Order | Family |
| --- | --- | --- | --- | --- | --- | --- | --- |
| U | PASV540 | | Amoebozoa | Lobosa | Tubulinea | Euamoebida | Hartmannellidae |
|  | ASV56 | | Archaea | Undefined | Undefined | Undefined | Undefined |
|  | PASV548 | | Amoebozoa | Lobosa | Undefined | Undefined | Undefined |
|  | BASV9 | | Bacteria | Proteobacteria | Undefined | Undefined | Undefined |
| UP | PASV111 | | Stramenopiles | Sagenista | Labyrinthulomycetes | Labyrinthulida | Labyrinthulaceae |
|  | PASV31 | | Amoebozoa | Lobosa | Tubulinea |  |  |
|  | PASV1204 | | Rhizaria | Cercozoa | Filosa-Granofilosea | Filosa-Granofilosea_X | Novel-Gran-234 |
|  | PASV634 | | Amoebozoa | Lobosa | Tubulinea | Leptomyxida | Flabellulidae |
|  | ASV57 | | Archaea | Undefined | Undefined | Undefined | Undefined |
|  | ASV28 | | Archaea | Undefined | Undefined | Undefined | Undefined |
|  | ASV20 | | Archaea | Crenarchaeota | Undefined | Undefined | Undefined |
|  | BASV1 | | Bacteria | Proteobacteria | Betaproteobacteria | Nitrosomonadales | Undefined |
| UD | ASV17 | | Archaea | Undefined | Undefined | Undefined | Undefined |
|  | ASV7 | | Archaea | Undefined | Undefined | Undefined | Undefined |
|  | ASV24 | | Archaea | Undefined | Undefined | Undefined | Undefined |
|  | PASV23 | | Stramenopiles | Sagenista | Labyrinthulomycetes | Labyrinthulida | Labyrinthulaceae |
|  | PASV393 | | Rhizaria | Cercozoa | Filosa-Sarcomonadea | Glissomonadida | Allapsidae |
|  | ASV5 | | Archaea | Undefined | Undefined | Undefined | Undefined |
|  | ASV9 | | Archaea | Undefined | Undefined | Undefined | Undefined |

**Supplementary Table 3.** The closest species to unidentified AOA and AOB in key taxa and their potential functions related to nitrogen cycling

| Treatments | ASV ID | Closest species | Accession ID | Similarity | Function related to N cycling | References |
| --- | --- | --- | --- | --- | --- | --- |
| U | BASV9 | Nitrosospira sp. Nsp17 | AY123825.1 | 100% | Nitrification | Liang et al., (2022) |
|  | ASV56 | Nitrososphaerota  archaeon | MK978758.1 | 99% | Ammonia oxidization; Obtain energy; Chemoautotrophy | Wei et al., (2023) |
| UP | ASV57 | Candidatus Nitrosocosmicus arcticus | MK978763.1 | 100% | Ammonia oxidization;  Heterotrophic metabolism | Alveset et al., (2019) |
|  | ASV28 | Nitrososphaerota  archaeon | MK978758.1 | 99% | Ammonia oxidization; Obtain energy; Chemoautotrophy | Wei et al., (2023) |
|  | ASV20 | Nitrososphaerota  archaeon | MK978758.1 | 99% | Ammonia oxidization; Obtain energy; Chemoautotrophy | Wei et al., (2023) |
| UD | ASV17 | Nitrososphaerota  archaeon | MK978758.1 | 99% | Ammonia oxidization; Obtain energy; Chemoautotrophy | Wei et al. (2023) |
|  | ASV7 | Nitrososphaerota  archaeon | MK978758.1 | 99% | Ammonia oxidization; Obtain energy; Chemoautotrophy | Wei et al. (2023) |
|  | ASV24 | Candidatus Nitrosocosmicus franklandus | KU290366.1 | 99% | Ammonia oxidization;  Energy generation | Nicol et al. (2019) |
|  | ASV5 | Nitrososphaerota  archaeon | MK978758.1 | 99% | Ammonia oxidization; Obtain energy; Chemoautotrophy | Wei et al. (2023) |
|  | ASV9 | Nitrososphaerota  archaeon | MK978758.1 | 99% | Ammonia oxidization; Obtain energy; Chemoautotrophy | Wei et al. (2023) |

**References**

Alves, R. J. E., Kerou, M., Zappe, A., Bittner, R., Abby, S. S., Schmidt, H. A., et al. (2019). Ammonia oxidation by the arctic terrestrial thaumarchaeote Candidatus Nitrosocosmicus arcticus is stimulated by increasing temperatures. *Front. Microbiol.*10, 1571. doi: 10.3389/fmicb.2019.01571

Liang, D., and Bowatte, S. (2022). Seed endophytic ammonia oxidizing bacteria in Elymus nutans transmit to offspring plants and contribute to nitrification in the root zone. *Front. Microbiol.* 13, 1036897. doi: 10.3389/fmicb.2022.1036897

Nicol, G. W., Hink, L., Gubry-Rangin, C., Prosser, J. I., and Lehtovirta-Morley, L. E. (2019). Genome Sequence of “Candidatus Nitrosocosmicus franklandus” C13, a terrestrial ammonia-oxidizing archaeon. *Microbiol. Resour. Ann.* 8(40), e00435-19. doi: 10.1128/MRA.00435-19

Wei, T. S., Gao, Z. M., Gong, L., Li, Q. M., Zhou, Y. L., Chen, H. G., et al. (2023). Genome-centric view of the microbiome in a new deep-sea glass sponge species Bathydorus sp. *Front. Microbiol*. 2023, 14: 1078171. doi: 10.3389/fmicb.2023.1078171
